# Massive Infected Dentigerous Cyst in a Young Child

**DOI:** 10.7759/cureus.39621

**Published:** 2023-05-28

**Authors:** Emmanuel D Azariah, Suganya Ramalingam, Thamizhchelvan Harikrishnan, Rachael J Khodabux

**Affiliations:** 1 Oral and Maxillofacial Surgery, Sri Ramachandra Dental College & Hospital, Sri Ramachandra Institute of Higher Education and Research, Chennai, IND; 2 Oral Pathology, Sri Ramachandra Dental College & Hospital, Sri Ramachandra Institute of Higher Education and Research, Chennai, IND

**Keywords:** bismuth iodoform paraffin pack, cholesterol clefts, carnoy's solution, unerupted tooth, dentigerous cyst

## Abstract

Dentigerous cysts are odontogenic cysts that form on the crown of an unerupted or partially erupted tooth. They are specifically anchored to the cementoenamel junction. Dentigerous cysts are known to rarely involve impacted deciduous teeth. Because of this rarity, this article reports a unique case of a five-year-old female patient who developed a dentigerous cyst in relation to a developing permanent left mandibular first molar tooth with its surgical treatment and histopathological features.

## Introduction

Dentigerous cysts are developmental odontogenic cysts that arise from the layer of reduced enamel epithelium to enclose the crown of an unerupted or partially erupted tooth at the cementoenamel junction. These are the second most prevalent kind of odontogenic cysts, accounting for 49% of all cystic lesions. The frequency of dentigerous cysts in children has been reported to be very low in literature. This article discusses a unique case in point of a five-year-old female patient who developed a dentigerous cyst, emphasizing its conservative treatment and histological findings.

Odontogenic cysts are formed from the epithelium involved in the formation of the dental apparatus and are classified into two types: developmental (keratocyst and dentigerous cysts) and inflammatory (radicular cysts) [1]. “Dentigerous” literally means “tooth-bearing.” These cysts develop when fluid accumulation occurs between the follicle and crown of an unerupted tooth or impacted teeth [2].

These cysts occur between five and 57 years of age, with a male predilection, often presenting as asymptomatic, and are diagnosed during routine radiographic examination most commonly in the mandibular third molar region [3]. Dentigerous cyst formation is estimated at around 1.44 in every 100 unerupted teeth [4]. Swelling, mild discomfort, mobility of adjacent teeth, and displacement may occur if the cyst enlarges to a more significant size (>2 cm in diameter) [5].

Dentigerous cysts usually present with a nonkeratinized-stratified squamous epithelium with occasional elongated interconnecting rete ridges in histopathology [2]. Mucous, ciliated, and occasionally sebaceous cells can be seen in these cysts. The presence of inflammation is frequently present with normal findings, and it must be distinguished from an odontogenic keratocyst [2]. The therapeutic method is determined by the size and location of the cyst, as well as the patient's age, afflicted dentition, and approximating to adjacent vital tissues [6]. The prognosis is excellent either with enucleation or marsupialization and recurrence is seldom noted [1,6].

## Case presentation

**Figure 1:**
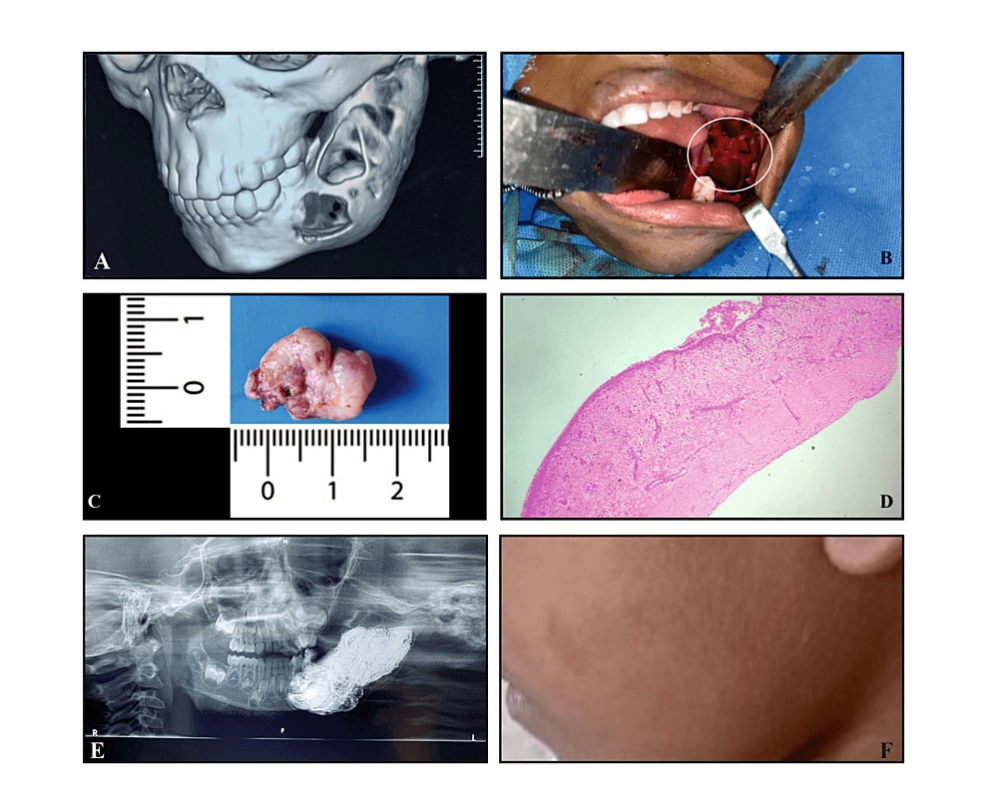
(A) Three-dimensional image revealed a huge single expansile osteolytic lesion associated with an impacted tooth showing a thinned-out cortex. (B) Intraoperative view of the cystic cavity. (C) The excised specimen in toto. (D) Photomicrograph of the lesion showing the cystic lesion lined by nonkeratinized stratified squamous epithelium (H and E: 4x view). (E) One-week postoperative orthopantomogram. (F) Clinical photograph of one-year follow-up.

**Figure 2:**
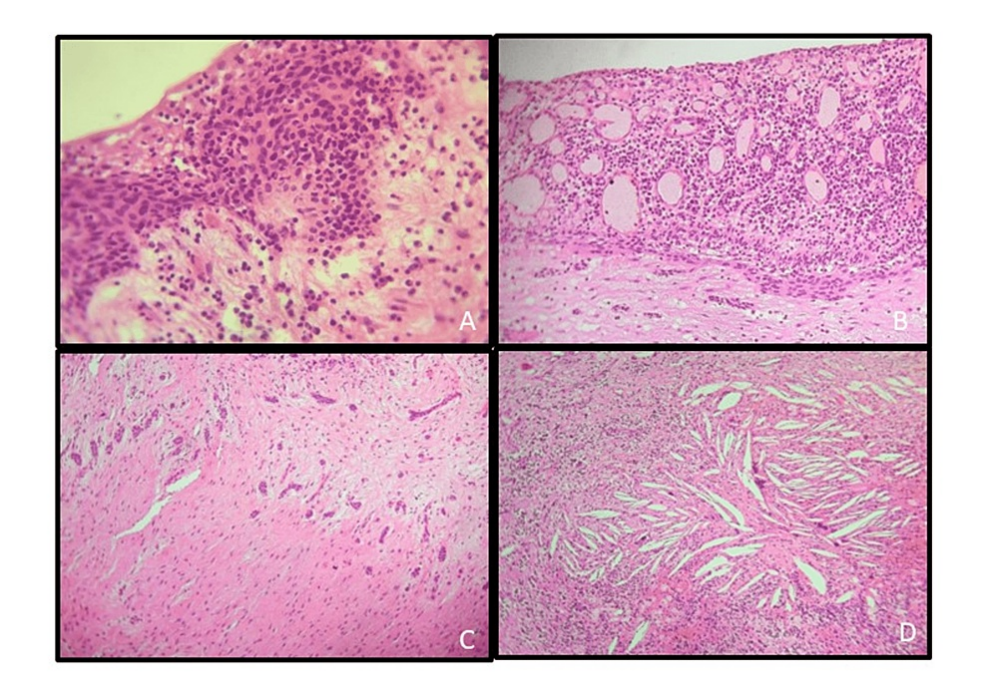
(A) Photomicrograph showing rete ridges developing into the connective tissue (hematoxylin & eosin (H&E): 10x view). (B) Photomicrograph showing scattered mucus cells in the cystic epithelium (H&E: 10x view). (C) Photomicrograph showing odontogenic cell rests in the connective tissue stroma (H&E: 4x view). (D) Photomicrograph revealing connective tissue stroma showing severe chronic inflammatory cells and cholesterol clefts (H&E: 10x view).

A preschool girl child visited the outpatient department of a private dental college with a chief complaint of swelling in the left lower back tooth region for the past month. Three-dimensional imaging (Figure 1A) revealed a huge single expansile osteolytic lesion associated with impacted teeth showing a thinned-out cortex. Due to the patient's age, invasive procedures were deferred and a valid diagnosis of odontogenic keratocyst or unicystic ameloblastoma was attained based on radiographic appearance. An excisional biopsy after enucleation of the lesion was planned under general anesthesia (GA). Under GA, the cystic lesion was aspirated and revealed a serosanguinous fluid. A crestal incision was made and the primary molar was extracted and the mucoperiosteal flap was elevated to reveal the thinned-out cortex (Figure 1B). After removing the thinned-out cortex, the cyst was carefully removed in toto (Figure 1C). Curettage and dissection with peanut gauze were used to enucleate the lesion. The permanent molar tooth bud was removed along with the lesion and the inferior alveolar nerve was found intact on the floor. Carnoy’s solution was used to cauterize the area to prevent a recurrence chemically. The lesion was closed directly with a bismuth iodoform paraffin pack. Formalin-fixed hematoxylin and eosin-stained sections histopathologically revealed a thin delicate nonkeratinized stratified squamous epithelium with focal areas of rete ridges proliferating into the connective tissue stroma. Scattered mucous cells were also seen in the epithelial lining. The underlying connective tissue revealed loosely arranged collagen fibers in a myxoid stroma, diffuse chronic inflammatory cell infiltrate areas of extravasated RBCs, odontogenic cell rests, and cholesterol clefts. Based on the histopathological correlation, a diagnosis of an infected/inflamed dentigerous cyst was given (Figure 1D and Figure 2). The patient's immediate and one-year follow-ups revealed no recurrence (Figures 1E, 1F).

## Discussion

Dentigerous cysts are odontogenic cysts that originate from the separation of the follicle from the crown of an unerupted tooth [7]. These cysts mostly occur in the mandibular third molar region followed by the maxillary third molar region, maxillary canine, and mandibular second premolar region [8]. Bilateral and multiple cysts are usually found in association with syndromes including cleidocranial dysplasia, Maroteaux-Lamy syndrome, and mucopolysaccharidosis. In the absence of these syndromes, the occurrence of multiple dentigerous cysts is rare[9]. The first molar being the first permanent teeth to erupt is usually not impacted and is not commonly associated with a dentigerous cyst.

Histopathogenesis of dentigerous cysts is based on intra-follicular and extrafollicular theories [10]. Main's theory suggests that the impacted tooth exerts pressure on the follicle, which ultimately causes the separation of the crown from the follicle with or without reduced enamel epithelium [10]. The osmolality of the cyst fluid is modified by increased permeability to glycosaminoglycans such as hyaluronic acid, heparin, and chondroitin sulfate, which causes rapid growth [11].

Dentigerous cysts are more common in the second and third decades with the usual occurrence between five and 57 years old. Here, we present a unique case of dentigerous cysts in a five-year-old female [12]. Generally, these cysts are often asymptomatic and are discovered as an incidental finding or when the patient presents with acute inflammation or swelling. The present case presented with a swelling of one-month duration. Radiographically, they are of three variants, i.e., central, lateral, and circumferential types [12]. Some lesions can grow up to 5 cm in diameter in three to four years. The larger lesions are associated with cortical expansion and erosion. It was not observed in the present case. These cysts are considered aggressive when they have a tendency to cause root resorption of adjacent teeth.

Dentigerous cysts can be either developmental or inflammatory in nature, with the former being the most prevalent [13]. Histopathologically, the noninflamed dentigerous cyst exhibits a thin, nonkeratinized epithelial lining and a fibrous connective tissue wall, which is loosely arranged, while the inflamed dentigerous cyst has a more collagenized fibrous wall with a variable infiltration of chronic inflammatory cells with the epithelium showing variable amounts of hyperplasia and rete ridges [3]. We observed a thin nonkeratinized stratified squamous epithelium in focal areas and proliferating epithelium developing into rete pegs were observed in other areas. The connective tissue stroma showed intense chronic inflammatory cell infiltration and the presence of cholesterol clefts.

These cysts are treated by enucleation along with the extraction of the unerupted tooth or marsupialization and have a remarkable prognosis. The abnormal position of the associated tooth and a large lesion, which seemed aggressive, justify enucleation, which made the entire lining available for histopathological examination. Odontogenic keratocyst, unicystic ameloblastoma, and dentigerous cyst are alternative diagnoses to be made when an impacted tooth is present, as observed on the radiograph, because they are more likely to develop in the mandibular molar region. To discriminate between the above lesions, a radiographic examination will not be sufficient, and conducting a histological examination will be required to distinguish such lesions [14].

## Conclusions

A dentigerous cyst is usually slow growing and is identified early. The age and the size of the lesion and its presentation were unusual in the present case. Though the age of the patient was favorable for a marsupialization, the abnormal position of the associated tooth and a large lesion that seemed aggressive justified enucleation, which made the entire lining available for the histopathological examination. The treatment modality may get complicated in pediatric patients due to factors such as continuous facial growth, a greater percentage of cancellous bone, increased bone turnover, and the presence of unerupted teeth. Since cysts can attain considerable size with minimal or no symptoms, early detection and removal of the cysts are important to reduce morbidity. It is also highly recommended that a regular post-surgical follow-up is necessary for these patients.
